# *Streptomyces alboflavus* RPS and Its Novel and High Algicidal Activity against Harmful Algal Bloom Species *Phaeocystis globosa*

**DOI:** 10.1371/journal.pone.0092907

**Published:** 2014-03-27

**Authors:** Bangzhou Zhang, Guanjing Cai, Haitao Wang, Dong Li, Xujun Yang, Xinli An, Xiaowei Zheng, Yun Tian, Wei Zheng, Tianling Zheng

**Affiliations:** State Key Laboratory of Marine Environmental Science and Key Laboratory of the Ministry of Education for Coast and Wetland Ecosystems, School of Life Sciences, Xiamen University, Xiamen, Fujian, China; University of Connecticut, United States of America

## Abstract

*Phaeocystis globosa* blooms have frequently occurred along coastal waters and exerted serious impacts on ecological environments by releasing toxic hemolytic substances, forming nuisance foam, and causing oxygen depletion. An actinomycete strain RPS with high algicidal activity against *P. globosa* was isolated and identified as *Streptomyces alboflavus*, based on morphology, physiological and biochemical characteristics, and 16S rDNA sequence analysis. RPS lysed 95% of *P. globosa* within 48 h by releasing an extracellular active substance into the growth medium. The activity of RPS supernatant was sensitive to temperature at and above 50°C and insensitive to pH from 3 to 11. The molecular weight of the active substance was between 100 Da and 1000 Da, and approximately 90% of it was extracted by ethyl acetate. It was presumed that the active component efficiently inhibited the movement of *P. globosa*, caused the flagella to fall off the algae, and finally lysed the algal cells. RPS showed a wide target range against harmful algae. *S. alboflavus* RPS with high algicidal activity and such novel features of temperature and pH sensitivity, low molecular weight, algicidal process, and target range possesses great potential in the biological control of *P. globosa* blooms.

## Introduction

Harmful algal blooms (HABs) have become serious marine environmental disasters, and are spreading all around the world, threatening marine organisms and human health, and limiting economic development in fisheries, aquaculture, and tourism [1]–[3]. Due to these severe negative effects, a number of strategies have been proposed to control HABs, involving ultraviolet light [4], microwave [5], clay [6], modified sand [7], plants [8], protozoan [9], and Chinese traditional medicines [10]. However, only a few of these are feasible and applicable in case of emergencies due to the high cost or side effects [6], [11]. In recent years, microbial agents mitigating HABs, especially naturally occurring algicidal bacteria [12]–[15] have attracted global attention.

Algicidal bacteria play a potentially important role in regulating the growth, metabolism, and toxin production of harmful algae [16], [17]. Factually, relationships between algicidal bacteria and harmful algae are quite complex and have been extensively researched [16], [18], of which the most conspicuous and important is the inhibition or lysis of harmful algae by algicidal bacteria. Consequently, plenty of algicidal bacteria which mainly belong to genera *Pseudoalteromonas*, *Alteromonas*, *Vibrio*, *Cytophaga*, and *Saprospira* were isolated [19]. The negative effects of algicidal bacteria on harmful algae are therefore considered as the basic principle of microbial methods to control HABs. The mode of action of algicidal bacteria can be divided into direct attack and algicide release. Direct attack requires attachment of bacteria to the harmful algae in order to make direct lysis, while algicide release mode is that bacteria release freely diffusible algicides, such as protein [20], amino acid [12], or alkaloid [21], to kill algal cells. With regard to the relationships, another aspect is that some harmful algae may inhibit or lyse algicidal bacteria [22]–[24], while some may supply nutrition to algicidal bacteria [25]. What is more special is that the co-culturing of algae with bacteria can enhance the production of algicidal compounds derived from algae themselves [26]. Besides these interactions above, bacteriophages are supposed to be involved in the relationships, through lysing algicidal bacteria to trigger the growth of harmful algae [27]. Presently, the major targeted harmful algae that have been investigated are dinoflagellates, diatoms, and cyanobacteria [16], [18], [28].

*Phaeocystis globosa*, a eukaryotic HABs-causing species, is reported to be associated with shellfish mortality [29], acid rain [30], and serious impacts on the whole ecological environment by releasing toxic hemolytic substances [31], forming nuisance foam [32], and causing oxygen depletion [29]. Meanwhile, the extracellular polymeric substances of *P. globosa* were also involved in the stability of quantum dots (toxic nanoparticles) in marine environments [33]. *P. globosa* blooms have frequently occurred along the southern coast of China [34], the eastern English Channel [35], and the South Central coast of Viet Nam [36]. However, only a few studies have focused on the interactions between *P. globosa* and microbes (bacteria [37] and viruses [38]) in recent years. Even though actinomycetes are commonly known for production of bioactive compounds, a few of algicidal actinomycetes and their algicidal activity against *P. globosa* were reported in attempt to develop potential microbial control methods [15].

In this study, we isolated an actinomycete strain RPS algicidal to *P. globosa* from Fujian Zhangjiangkou Mangrove National Nature Reserve in China. This actinomycete was identified as *Streptomyces alboflavus*. Furthermore, the algicidal mode, sensitivity of algicidal substance to temperature and pH, molecular weight range and polarity of the active substance, algicidal process of the ethyl acetate extract, and susceptible algae range were investigated to better understand the novel activity against *P. globosa*.

## Materials and Methods

### Ethics statement

No specific permissions were required for the mangrove sediment sampling stated in this study. The field study did not involve endangered or protected species, and the location is 23°53′–23°56′ N and 117°24′–117°30′ E.

### Algal cultures

*P. globosa* culture was obtained from State Key Laboratory of Marine Environmental Science (Xiamen University), along with cultures of *Phaeodactylum tricornutum*, *Asterionella japonica*, *Chlorella autotrophica*, *Nannochloropsis oceanica*, *Platymonas helgolandica*, *Platymonas subcordiformis*, *Dunaliella salina*, *Chlorella* sp., *Dicrateria inornata*, *Isochrysis galbana*, and *Heterosigma akashiwo*. Cultures of *Alexandrium tamarense* and *Scrippsiella trochoidea* were from the Algal Culture Collection, Institute of Hydrobiology, Jinan University, China. All algal cultures were maintained in sterilized f/2 medium at 20±1°C under a 12 h: 12 h light-dark cycle with a light intensity of 4000 lx. To test for algicidal activity, *P. globosa* was inoculated into a 24-well cell plate, and the fluorescence intensity (RFU) of algae in each well was measured at an excitation wavelength of 440 nm and emission wavelength of 680 nm (Spectra max M2, Molecular Devices Corporation) to be treated as biomass (linear relationship between RFU and biomass in Supporting data Figure S1).

### Isolation of algicidal actinomycetes

Sediment sample (0–20 cm) from Fujian Zhangjiangkou Mangrove National Nature Reserve, China was dissolved in sterilized seawater and shaken at a speed of 150 rpm for 1 h, followed by stillness for 1 h. The supernatant was diluted to 10^−1^, 10^−2^, and 10^−3^ levels. 100 *μ*L of each dilution was spread onto the agar plates (soluble starch 15 g L^−1^, NaNO_3_ 1 g L^−1^, K_2_HPO_4_ 0.5 g L^−1^, MgSO_4_•7H_2_O 0.5 g L^−1^, and FeSO_4_•7H_2_O 0.01 g L^−1^, dissolved in natural seawater for agar plates while dissolved in deionized water for liquid fermentation) and incubated for 7 d at 28°C. Colonies with distinct morphologies were further purified several times until single colony type was obtained and then stored at −80°C in 10% (v/v) glycerol.

One of the strains was white in color initially and became yellow-red in the following days, and it was named RPS (red pigment strain). RPS was grown in 50 ml of fermentation medium (28°C, 180 rpm) for 7 d. 100 *μ*L, 150 *μ*L, 200 *μ*L, and 300 *μ*L of RPS culture were inoculated into 1.8 mL of logarithmic phase culture of *P. globosa* (RFU approximately  = 300, 2.42×10^6^ cells mL^−1^, the similar RFU as follows), while 200 *μ*L of fresh medium was added to algal culture as a control. The fluorescence intensity and color of the algal cultures were monitored every 12 h. All experiments had three replications in this study. The algicidal ratio was calculated using the following formula:

Algicidal ratio (%) = (F_c_−F_t_) / F_c_×100. F_t_: fluorescence intensity of treated algal culture, F_c_: fluorescence intensity of algal culture as control.

### Identification of the algicidal actinomycete

Cultural morphologies of RPS on different media and the investigation of physiological and biochemical characteristics were carried out as previously described [39], [40]. For scanning electron microscope (SEM, JSM-6390, JEOL), samples were performed as follows: coverslips with RPS colony grown on Gause agar plate for 15 d were treated with 2.5% glutaraldehyde for 4 h, followed by 3 times of washing with 0.1 M PBS (pH = 7.4). Materials were dehydrated in 30%, 50%, 70%, 90%, 95%, and 100% serial ethanol-water solutions, and finally stored in pure tertiary butanol at 4°C overnight. The coverslips were lyophilized and sputter coated with gold (JFC-1600, JEOL) for SEM observation.

The genomic DNA of RPS was extracted as previously described [13], with additional microwave (850 W) treatment for 30 s. The 16S rRNA gene was amplified using primers 27F (5′-AGAGTTTGATCCTGGCTCAG-3′) and 1492R (5′-GGTTACCTTGTTACGACTT-3′). PCR products were purified and transformed into *Escherichia coli* DH5*α* competent cells and the clones with a 1.5 kb insert were sequenced (Invitrogen Biotechnology Co., Ltd.). The sequence was submitted to GenBank and BLAST to get related sequences. All sequences were aligned by CLUSTALX 2, and a phylogenetic tree was constructed using MEGA4 software by Neighbor-Joining method with bootstrap analysis of 1000 replicates [41].

### Algicidal mode of RPS

In order to determine whether the algicidal activity of RPS was from an extracellular substance or the bacterium itself, 4 treatments were carried out with RPS culture that had been incubated for 7 d (Effect of fermentation time on the algacidal activity in Supporting data Figure S2): (a) 1 mL of RPS culture was centrifuged at 9131 *g* (10000 rpm, Eppendorf-5424R) for 5 min, and the supernatant was transferred to a new tube (Supernatant); (b) The precipitate was washed 3 times and re-suspended with 1 mL of fresh fermentation medium (Mycelia); (c) 1 mL of RPS culture without any treatment (Fermentation broth); (d) 1 mL of fresh fermentation medium was used as control. 200 *μ*L of the above treatments were added into separate 1.8 mL of algal culture (10% as final concentration, the same below) and monitored the RFU after incubation for 48 h to calculate the algicidal ratio (the same below).

### Sensitivity of algicidal activity to temperature and pH

The supernatant of RPS culture in 4.5 mL microcentrifuge tubes was incubated in a water bath at 30, 40, 50, 60, 80, and 100°C for 2 h and then cooled to room temperature. The pH of RPS supernatant was adjusted to 3, 5, 7, 9, and 11 for 2 h and then adjusted back to the initial pH. Treated samples were added into the algal culture at 10% ratio, as well as the supernatant without any treatments and equivalent of fresh medium used as controls.

### Molecular weight range and polarity of algicidal substance

For molecular weight range, the initial supernatant was loaded into dialysis bags with molecular weight cut-offs (MWCOs) of 100, 500, and 1000 Da, followed by dialysis for 48 h in fresh medium which was replaced every 24 h. The dialyzed supernatants, initial supernatant, and fresh medium were added into algal culture at 10% ratio. To understand the polarity, supernatant (50 mL) was extracted 3 times with different organic solvents (n-hexane, benzene, chloroform, ethyl acetate, and n-butanol) at ratio of 1∶1 (v:v). The same organic phase was transferred together. As regards to methanol, the supernatant (50 mL) was evaporated at 35°C by rotary evaporator and then extracted. Each extract was vacuum evaporated and then dissolved in 2.5 mL of DMSO. 10 *μ*L of each extract (equal to 200 *μ*L of supernatant in algicidal substance dose, no loss considered) was added into 1.99 mL of algal culture. The fresh medium was extracted with ethyl acetate to show the effects of concentrating components of fresh medium (Supporting data Figure S3).

### Observation of algicidal process

Ethyl acetate extract (10 *μ*L, approximately 100 *μ*g) was added into 1.99 mL of algal culture. The culture was sampled at 4, 12, 24, 36, and 48 h after treatment. These samples were centrifuged at 5000 *g* for 5 min, and the precipitate cells were fixed by 200 *μ*L of 2.5% glutaraldehyde for 4 h. The samples were washed and dehydrated using the same methods as subsection 2.4. Coverslips were observed by SEM (JSM-6390, JEOL).

### Susceptibility of algae to RPS

The susceptibility of algae to RPS was tested, including haptophyte *P. globosa*, raphidophyte *H. akashiwo*, dinoflagellates (*A. tamarense*, *S. trochoidea*), diatoms (*P. tricornutum*, *A. japonica*), green algae (*C. autotrophica*, *N. oceanica, P. helgolandica*, *P. subcordiformis*, *D. salina*, *Chlorella* sp.), and golden algae (*D. inornata*, *I. galbana*). 10 *μ*L of ethyl acetate extract was added to each algal culture (2 ml, RFU approximately = 300, cell concentration in Supporting data Table S1). Addition of 10 *μ*L of DMSO was used as a control.

### Statistical analysis

Data points are presented as mean ± standard error of triplicate assays. One-way ANOVA and post-hoc Turkey test were employed to determine the significant difference between groups (*α* = 0.05). Two-way ANOVA followed by Turkey test was used to analyze the interaction effect of RPS culture concentration and time. Differences were considered statistically significant when P<0.05. Linear regression was performed to show the linear relationship between cell number and RFU. All statistical analysis was performed with SPSS 18.0 (SPSS Inc.; Chicago, IL).

## Results

### Isolation of algicidal actinomycete RPS

**Figure 1:**
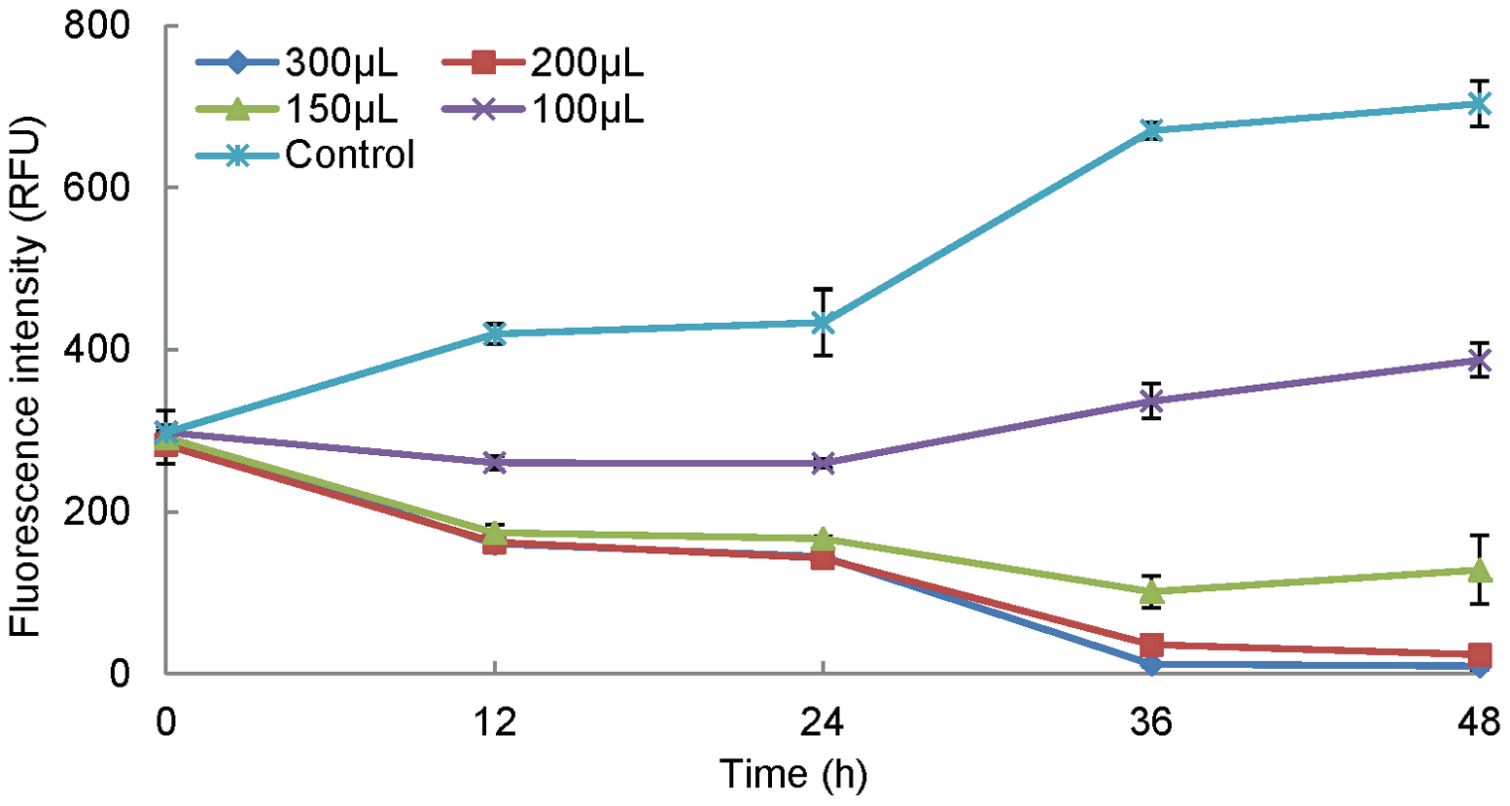
Algicaidal effect of RPS supernatant on *P. globosa* at different time points. Data points: mean ± standard deviation of triplicate assays. Error bars not visible are smaller than symbols. The algal biomass treated with RPS supernatant decreased dramatically in 48 h. A significant interactive effect of RPS supernatant and time was also observed by analysis of two-way ANOVA (P<0.001).

The strain named RPS was isolated from the sediment of Fujian Zhangjiangkou Mangrove National Nature Reserve. Algicidal activity of RPS was tested in a 24-well cell plate (Figure 1). This figure indicated that the biomass of the algal culture treated with 150 *μ*L, 200 *μ*L, and 300 *μ*L of RPS decreased dramatically in 48 h, while the control grew normally. A significant interaction effect of RPS supernatant and time (P<0.001) was also observed by analysis of two-way ANOVA. Nearly all algal cells fell to the bottoms of the wells after 4 h. Combined with cells sinking, there was approximately an 100% algicidal ratio at 48 h which proves that strain RPS is a strong algicidal actinomycete against *P. globosa*. Since treatments with 200 *μ*L and 300 *μ*L of supernatant showed almost the same activity during 48 h (P>0.05), 200 *μ*L was chosen as the basic amount to determine the culture activity in different treatments in the following studies.

### Identification of algicidal actionmycete RPS

**Figure 2:**
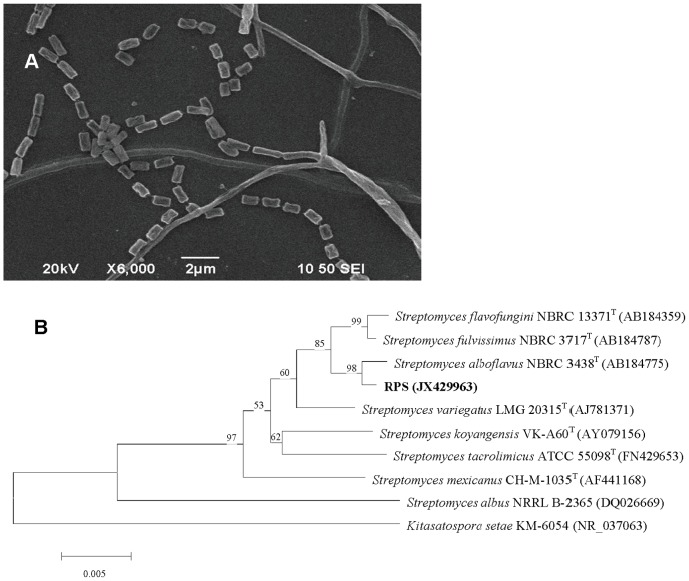
Morphology (A) and phylogenic tree (B) of RPS. Morphology from SEM results (JSM-6390, JEOL) and phylogenic tree based on 16S rDNA sequences by Neighbor-Joining analysis. *Kitasatospora setae* KM-6054 (NR_037063) was used as the out group.

The colonies of strain RPS on Gause synthetic agar No. 1 medium for 15 d were circular and dry. The substrate mycelia were abundant and golden-yellow, while aerial mycelia were rare. No soluble pigments were released into the medium. Spores were cylinder with a smooth surface (Figure 2A). Typically, all these characteristics belong to *Streptomyces*. Cultural characteristics of aerial mycelia, substrate mycelia, and soluble pigments on usual media were similar with each other (Table 1). Substrate mycelia were abundant and yellow or red in color, while the aerials were poor and in pale color (white or light pink). No pigments were released by RPS growing on all media except potato dextrose agar (light yellow).

**Table 1:** Culture characteristics of RPS at 28°C on different media.

Media	Aerial mycelia	Substrate mycelia	Pigments
Gause synthetic agar No. 1	Pink-white	Golden yellow	None
Mineral salt starch agar	Light pink	Orange	None
Clinton synthetic agar No. 1	Pink-white	Melon-pink	None
Czapek's agar	Light cherry red	Rose red	None
Glucose asparagine	White	Camellia red	None
Potato dextrose agar	Light pink	Golden yellow	Light yellow
Emerson agar	Bean yellow	Rice yellow	None
Yeast-malt extract agar	Pink-white	Light brown	None

In terms of physiological and biochemical characteristics, RPS was capable of liquefaction of gelatin, peptonization of milk, and hydrolysis of starch, and able to use glucose, sucrose, glycerol, galactose, mannitol, and xylose as the sole carbon and energy source, but could not utilize cellulose, lactose, or maltose to grow.

The 16S rRNA gene of RPS was amplified, and an approximately 1.5 kb band was observed by 1% agarose electrophoresis. The obtained sequence (GenBank accession number JX429963) showed that the most probable affiliation of RPS was to the genus *Streptomyces* by BLAST, and the strains with similarity of more than 98% were all streptomycetes. The phylogenetic tree (Figure 2B) produced by the neighbor-joining analysis revealed that the closest relative of RPS was the strain *S. alboflavus* NBRC 3438^T^ with the similarity of 99.7%, and all streptomycetes were classified into the same branch.

RPS was very similar to *S. alboflavus* in morphological, physiological, and biochemical characteristics, including poor aerial mycelia, yellow to red substrate mycelia, no soluble pigments released, potential of liquefaction of gelatin, peptonization of milk, and hydrolysis of starch [42]. In further consideration of the close phylogenetic relationship to *S. alboflavus*, strain RPS was identified as *Streptomyces alboflavus* (*S. alboflavus* RPS).

### Algicidal mode

**Figure 3:**
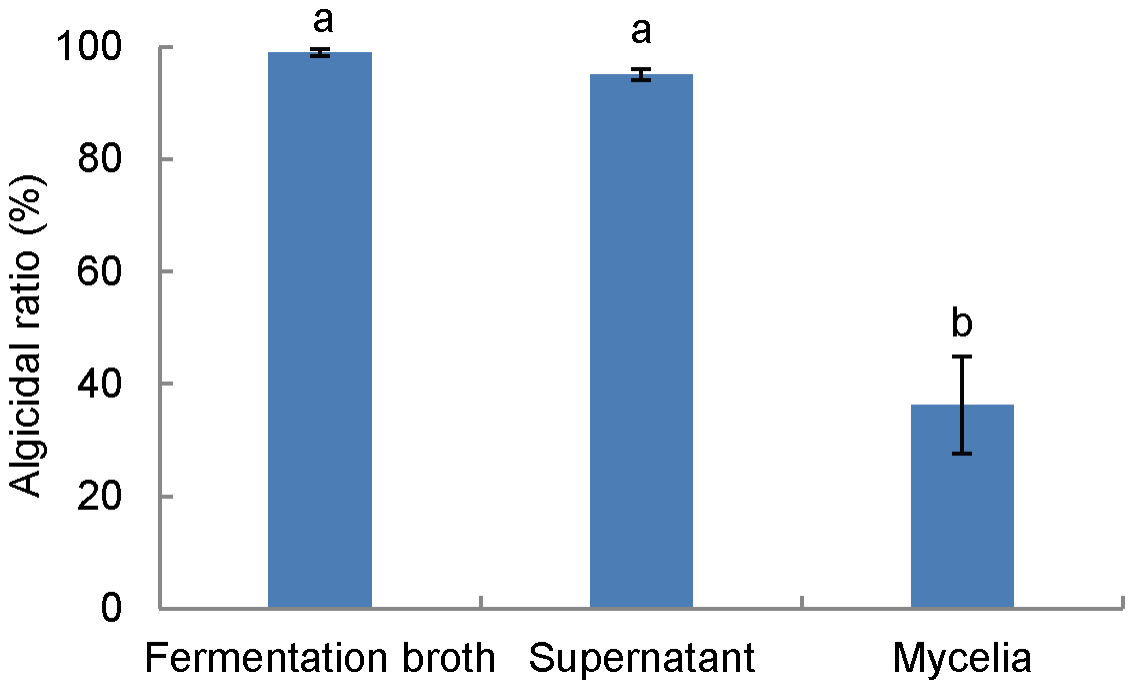
Algicide release mode of RPS against *P. globosa*. Data from RFU assay of algicidal effect (1∶10, v:v). Data points: mean ± standard deviation of triplicate assays. Treatment means with different letters differed significantly (P<0.001), analyzed using one-way ANOVA.

Algicidal activities of initial fermentation broth, supernatant, and mycelia were investigated. The algicidal ratios of the initial broth and supernatant reached 99% and 95% (a same active level P>0.05), respectively, while mycelia showed a much lower ratio of approximately 36% (P<0.001, Figure 3). The significant difference (P<0.001) of the algicidal activity from this comparison shows that RPS exhibits the algicidal activity in an indirect way by releasing an extracellular active substance into the growth medium. The partial activity in the mycelia treatment may be caused by some active substance released by mycelia after being added into the algal culture.

### Sensitivity of algicidal activity to temperature and pH

**Figure 4:**
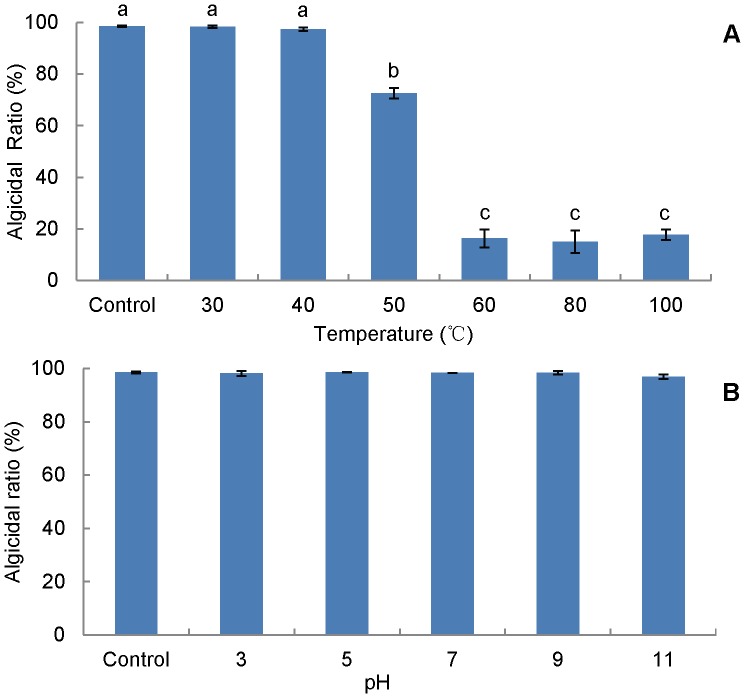
Sensitivity of supernatant algicidal activity to temperature (A) and pH (B). Data from RFU assay of algicidal effect (1∶10, v:v). Control represents the initial fermentation broth without any treatments. Data points: mean ± standard deviation of triplicate assays. Temperature treatment means with different letters in (A) differed significantly (P<0.001) by analysis of one-way ANOVA, suggesting that the algicidal activity of RPS is sensitive to temperature, while no significant difference (P>0.05) was observed among treatments with pH in (B), meaning that the activity is insensitive to pH.

The supernatant exhibited different trends in treatments with different temperatures and pH for 2 h. The activity of the supernatant was very sensitive to temperature, as it declined significantly (p<0.001) by 25% and 80% when the temperature was raised to 50°C and 60°C (and above), respectively, compared with the untreated supernatant (Figure 4A). However, the activity was resistant to pH, as the supernatant was similar active (p>0.05) in a pH range of 3–11 (Figure 4B). Hence, to keep the activity in the supernatant, more attention should be paid to the steps related to temperature changes.

### Molecular weight range and polarity of algicidal substance

**Figure 5:**
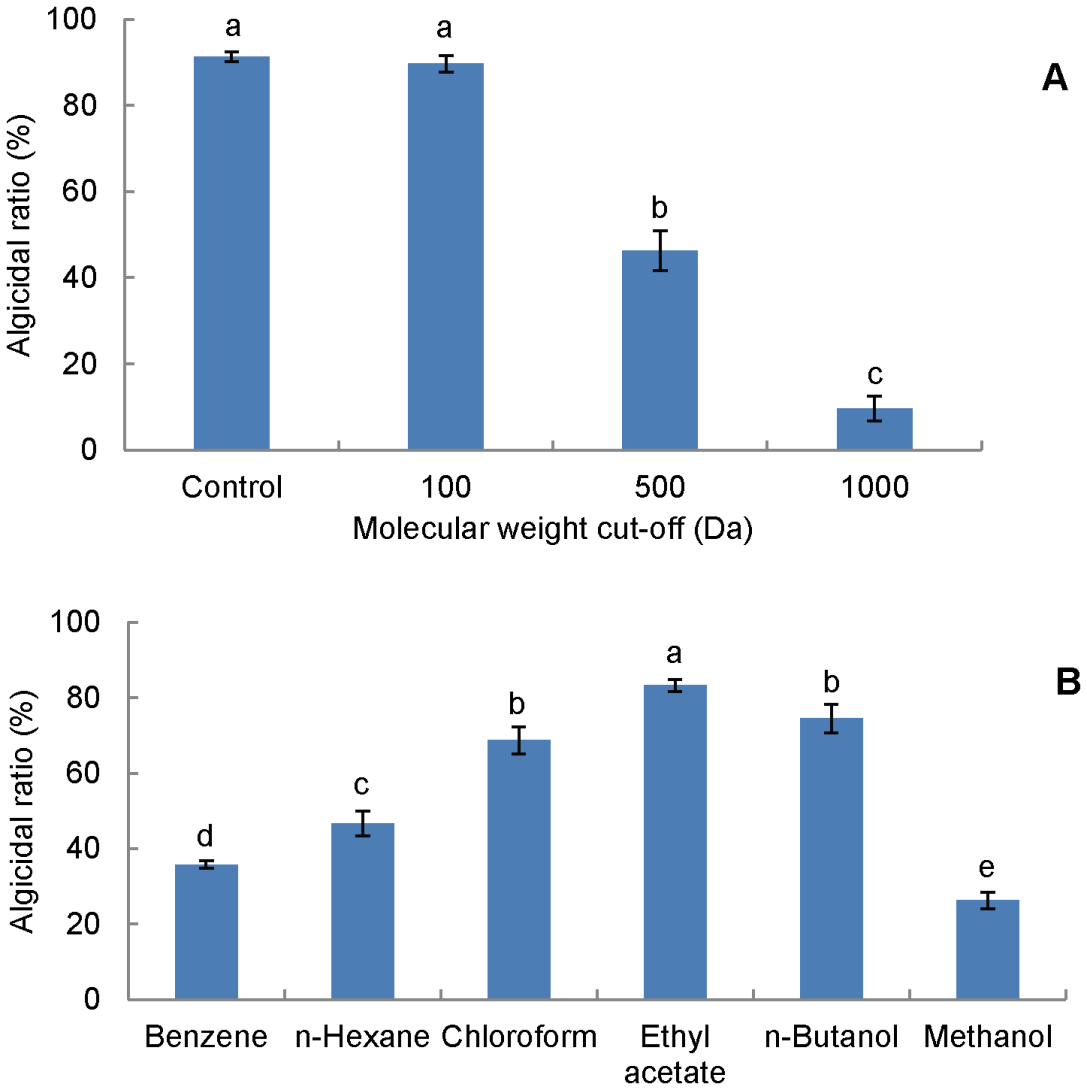
Algicidal activity of initial supernatant and dialyzed supernatants (A) and different organic solvent extracts (B). Data from RFU assay. Data points: mean ± standard deviation of triplicate assays. In (A), initial supernatant (Control) and dialyzed supernatants were added at 10% ratio, and treatment means with different letters differed significantly (P<0.001) by analysis of one-way ANOVA. In (B), 10 *μ*L extract was added into 1.99 mL algal culture (equal to 1∶10 of v:v in algicidal substance dose, no loss considered during extraction), and treatment means with different letters differed significantly (P<0.05) by analysis of one-way ANOVA.

The algicidal activities of dialyzed supernatants were significantly different (p<0.001) from each other (Figure 5A). The figure showed similar activities (p>0.05) in the initial (91%) and dialyzed supernatant with a cut-off of 100 Da (89%), but dramatically decreased activity (p<0.001) in the dialyzed supernatant with a cut-off of 1000 Da (10%). Certain reduction (p<0.001) was also observed in dialyzed supernatant with a cut-off of 500 Da (not as dramatic as that of 1000 Da). Therefore, it is credible to speculate that the molecular weight of the active substance ranges between 100 Da and 1000 Da.

The polarity of the active substance was investigated through the comparison of activities of different organic extracts (Figure 5B), of which the ethyl acetate extract possessed the highest activity (83%). Other solvents, which were more or less polar than ethyl acetate, led to lower extraction efficiencies (p<0.05). These results imply that the active substance should be a low polar and hydrophobic molecule with a low molecular weight. As the extract amount (10 *μ*L) added in this experiment was equal to 200 *μ*L of initial culture with no loss considered during extraction, ethyl acetate was able to extract approximately 90% of the active substance in the supernatant.

### Algicidal process

**Figure 6:**
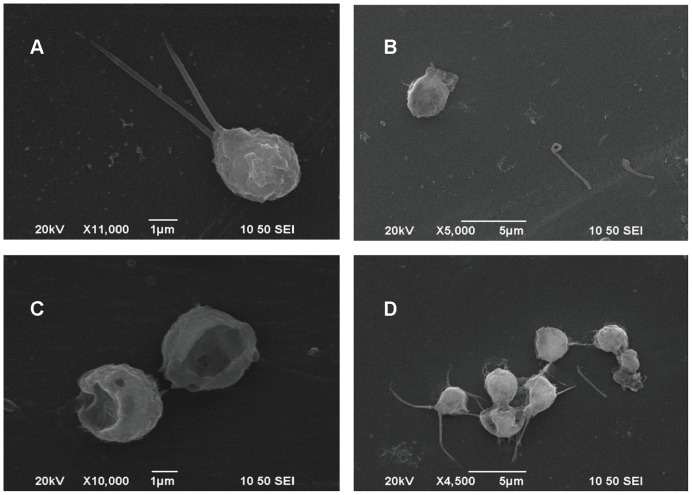
Morphological changes of *P. globosa* treated by ethyl acetate extract from RPS supernatant within 48 h. (A) normal *P. globosa*, (B) flagella falling off the alga, (C) broken cells, and (D) algae at different damaged levels. The putative algicidal process is that the algicidal substance inhibits the movement of *P. globosa*, then causes the flagella to fall off the algae, and finally lyses the algal cells.

Nearly all algal cells fell to the bottoms after 4 h treatment with ethyl acetate extract, and the precipitated algal cells faded gradually along with time. The whole algal culture was clear almost without color at 48 h. Based on the SEM observation and optical observation of several time-point samples, the algicidal process was inferred as follows. A normal *P. globosa* cell possesses two intact flagella (Figure 6A); the movement of alga was inhibited by the active component added, which even caused the flagella to fall off the algae (Figure 6B); the active component ruptured the algal cells, leaving broken cell walls (Figure 6C); as not all cells were sensitive to the active substance at the same level, the former three occasions might exist in the same field of vision (Figure 6D). Factually, cell lysis was also proved by the results in Figure 1, where the fluorescence intensity dropped dramatically over time.

### Susceptibility of algae to RPS

The ethyl acetate extract lysed 10 of the 14 tested algae (Table 2), which were raphidophyte *P. globosa*, yellow-green algae of *H. akashiwo*, green algae of Chlorophyta (*P. helgolandica, P. subcordiformis*), golden algae of Chrysophyta (*D. inornata*, *I. galbana*), dinoflagellates of Pyrrophyta (*A. tamarense, S. trochoidea*), and diatoms of Bacillariophyta (*P. tricornutum, A. japonica*). However, green algae of Chlorophyta (*C. autotrophica*, *N. oceanica, D. salina*, *Chlorella* sp.) were not lysed. These results indicate that RPS has a wide target range and certain specificity, since it had no effect on 4 tested green algae (Table 2).

**Table 2:** Susceptibility of algae to ethyl acetate extract of RPS supernatant.

Phylum	Species	Activity^a^	Flagellum
Haptophyta	*P. globosa*	+	Yes
Xanthophyta	*H. akashiwo*	+	Yes
Chlorophyta	*C. autotrophica*	−	No
	*N. oceanica*	−	No
	*Chlorella* sp.	−	No
	*P. helgolandica*	+	Yes
	*P. subcordiformis*	+	Yes
	*D. salina*	−	Yes
Chrysophyta	*D. inornata*	+	Yes
	*I. galbana*	+	Yes
Pyrrophyta	*A. tamarense*	+	Yes
	*S. trochoidea*	+	No
Bacillariophyta	*P. tricornutum*	+	No
	*A. japonica*	+	No

a +: algicidal ratio more than 60%, −: algicidal ratio less than 20%.

## Discussion

### Algicidal Streptomyces against harmful *P. globosa*

*Phaeocystis* blooms have occurred frequently around the world since its first recorded observation as early as 1923 [43]. These blooms exerted negative effects on higher trophic levels in the marine ecosystem, and consequently influenced human activities such as fisheries and coastal tourism [44]. Hence, it is urgent to investigate the interactions between microbes and *Phaeocystis*, and develop potential microbial control methods. In this study, we isolated and identified *Streptomyces alboflavus* strain RPS, and then focused on its strong algicidal function and novel algicidal properties against *P. globosa*. Indeed, several strains of actinomycetes have been reported to show antialgal activity against cyanobacteria [45] and dinoflagellates [46]. However, there have been a relatively few algicidal *Streptomyces* strains reported against *P. globosa*, including the strain of *Streptomyces* O4–6 as well as two bacteria belonging to another genus *Bacillus* [15], [47]. Therefore, the study of RPS will further widen the resource pool of antagonistic microbes to *P. globosa*, as well as other harmful algae like *A. tamarense* and *H. akashiwo* (Table 2).

### High algicidal activity of RPS

*S. alboflavus* RPS achieved a 95% algicidal ratio against *P. globosa* in a short time of 48 h (Figure 1), which proves RPS is a highly efficient algicidal streptomycete. Previous reports showed much weaker algicidal effects. For example, *Streptomyces* S-9 isolated by Yamamoto et al. [45] needed 5 d or more to achieve a stable effect on cyanobacteria. Also, Choi et al. [48] reported that the cyanobacterial biomass was just suppressed by 84.5% after 7 d treatment of *S. neyagawaensis*, and Yan et al. [47] showed that bacteria Y01 and Y04 could remove *P. globosa* in 6 d. In this study, the similar activity (p<0.001) in the initial broth and supernatant after centrifugation indicated that RPS exerted the algicidal activity through the algicide release mode (Figure 3). Although algicidal compounds have been investigated for decades, leading to identification of algicidal proteins [20], peptides [49], amino acids [12], antibiotics [15], pigments [50], alkaloids [21], and biosurfactants [51], difficulties still exist in the isolation and elucidation processes of the algicidal compound in this study (We got a fraction with 99% algicidal ratio at 20 *μ*g mL^−1^ through silica column chromatography, TLC, and HPLC). Algicidal compounds and their bioactive characteristics vary greatly between species of algicidal bacteria, requiring different and specific compound isolation procedures both in extraction and chromatography [52].

### Novel algicidal features

RPS showed several novel algicidal features. What should be first focused on is the sensitivity to temperature and pH (Figure 4). Treatments at 50°C and above resulted in vast loss (p<0.001) of activity of the supernatant (Figure 4A), which led us to think the active component could be a protein, as several previous algicidal proteins were reported to be susceptible to temperature. For example, Lee et al. [53] reported an extracellular protease from *Pseudoalteromonas* A28 against *Skeletonema costatum* and the protease activity was abolished by incubation at 68°C for 1 h or 100°C for 15 min. Also, Paul and Pohnert [54] reported an algicidal protease lost the activity against *S. costatum* in the condition of 80°C for 10 min. However, the stable activity (p>0.05) of RPS within the pH range of 3–11 (Figure 4B) implies that the active component may not be a protein, as active proteins are generally pH-sensitive, such as violaxanthin deepoxidase [55], potassium channel [56], and Glycoprotein G [57]. What's more is that the treatment with proteinase K did not reduce the activity of RPS supernantant (data not shown). This paradoxical evidence suggests that this strain possesses a unique active substance which is temperature-sensitive but not pH-sensitive.

The dialysis result revealed that the molecular weight of the active substance should range between 100 Da and 1000 Da (Figure 5A), meaning a small molecule. Recently, several small molecule algicides have been reported, including *β*-cyanoalanine [12], nigericin [15], harmane [21], and rhamnolipid biosurfactants [51]. As for the partially reduced activity (p<0.001) in dialysis bag of MWCO of 500 Da, it is possible that the molecular weight of the active component is close to 500 Da, meaning that some of the component could occasionally pass through the dialysis bag with the cut-off of 500 Da and cause the decrease in activity. Extraction by ethyl acetate recovered nearly 90% of active substance in the supernatant, and showed the highest activity (p<0.05, Figure 5B). This high efficiency indicates that ethyl acetate can be used to extract the active substance in late-stage study. In fact, organic solvents were often used to isolate algicidal compounds. For example, Sakata et al.[58] isolated the 2,3-indolinedione against *Chaetoceros ceratosporum* by ethyl acetate. Li and Hu [59] also reported an algicidal ethyl 2-methylacetoacetate separated by ethanol and chloroform.

Based on the sensitivity to temperature, insensitivity to pH, molecular weight range, and high extraction ratio by ethyl acetate, it is inferred that the active component should be a low polar substance with a low molecular weight. To avoid degradation or loss of the bioactive component [52], more attention should be focused on temperature during the subsequent isolation and conservation steps, including concentration of supernatants, evaporation of extracts and chromatography.

### Presumption of algicidal process

Through the SEM and optical observation, it is estimated that flagella movement of *P. globosa* was efficiently inhibited by the active substance in the ethyl acetate extract, resulting in decreased cell motility. Subsequently, the flagella fell off the cells, and these attacked cells were lysed (Figure 6). This presumption was confirmed by the fact that the algae were sunk in the first 4 h after the addition of the active substance under optical observation, and the amounts of flagella and broken cell walls were observed later under SEM. Additionally, most of the susceptible algae possess flagella, such as yellow-green algae *H akashiwo*, green algae *P. helgolandica* and *P. subcordiformis*, dinoflagellates *A. tamarense* and *S. trochoidea*, and golden algae *I. galbana* (Table 2), while the non-susceptible algae do not possess flagella, including *C. autotrophica, Chlorella* sp., and *N. oceanica* (Table 2), which may be an indirect proof that the active component targets the flagella. Although this presumption needs more supporting evidence to prove, it may be a novel direction to study the algicidal mechanisms focusing on the movement of flagella. This direction may hopefully break through the bottlenecks of the present algicidal mechanism studies that have been primarily focusing on the oxidative damage and antioxidant responses [60]–[62], such as CAT, MDA, SOD.

### Susceptible algae

RPS showed a wide algicidal range toward harmful algae. RPS was antagonistic to HABs-causing algae *P. globosa*, *H. akashiwo*, *A. tamarense*, *S. trochoidea*, and *A. japonica*, as well as some other algae *P. helgolandica*, *I. galbana*, etc. However, it was not active against *C. autotrophica* (fish bait alga), *D. salina* (fish bait alga), *N. oceanica* (potential biofuel alga), and *Chlorella* sp. (Table 2), which may be an advantage (no effect on beneficial algae) of RPS when used as a biological agent in control of HABs in the future [48]. Besides, *S. alboflavus* RPS possesses the great potential to produce industrial pigments when the culture supernatant is used as an algicidal agent, since there was a large amount of red matter that was separated from the ethanol acetate extract of RPS mycelia.

### Conclusion

A strong algicidal streptomycte RPS against harmful algae *P. globosa* was isolated and identified as *Streptomyces alboflavus*. Furthermore, the novel algicidal features of RPS were investigated and concluded as follows: (i) high algicidal efficiency of more than 95% algicidal ratio in 48 h; (ii) sensitivity to temperature at and above 50°C, but resistance to a pH range of 3–11; (iii) the molecular weight of active component ranging 100–1000 Da and approximately 90% extraction ratio by ethyl acetate; (iv) putatively algicidal process of inhibiting the movement of flagella, followed by lysis; (v) a wide target range against harmful algae *P. globosa*, *H. akashiwo*, *A. tamarense*, *S. trochoidea*, and *A. japonica*. RPS with the high and novel algicidal activity gives a hint on controlling *P. globosa* blooms by microbial methods and effectively extends the resource pool of algicidal microbes against harmful algae.

## Supporting Information

Figure S1 **Linearity between the cell number of** ***P. globosa*** **and fluorescence intensity (RFU).** Cell numbers were counted manually by optical microscope. RFU was measured at the excitation wavelength (λex) = 440 nm and emission wavelength (λem) = 680 nm (Spectra max M2, Molecular Devices Corporation). Data points: mean ± standard deviation of triplicate assays. The linear relationship was analyzed by linear regression (R^2^ = 0.990).
(TIF)

Figure S2 **Effect of fermentation time on algicidal activity of RPS supernatant against** ***P. globosa*** **.** Results showed that activities of the supernatants were higher at 6 d, 7 d and 8 d compared with those at early days, and decreased gradually over fermentation time, and 7d was chosen in this study. Data from RFU assay of algicidal effect (1∶10, v:v) of supernatant samples collected from RPS fermentation broth each day for 10 days. Data points: mean ± standard deviation of triplicate assays. Treatment means with different letters differed significantly (P<0.05) by analysis of one-way ANOVA. The wet weight and dry weight of the mycelia biomass in 100 mL fermentation broth for 7 d were approximately 0.77±0.06 g and 0.07±0.03 g, respectively.
(TIF)

Figure S3 **Algicidal activity comparison of ethyl acetate extract of fresh medium with that of fermentation supernatant.** Data from RFU assay of algicidal effect of 10 *μ*L extract added into 1.99 mL algal culture (the same ratio as the polarity test). Data points: mean ± standard deviation of triplicate assays. Since the algicidal ratio of extract of fresh medium is much weaker than that of supernatant extract (P<0.001) by analysis of one-way ANOVA, the weak effect can be ignored in this study.
(TIF)

Table S1 **Cell concentration of each algal species (RFU approximately  = 300) tested in this study.** The value represents the average of three replicates.
(DOCX)
