# Supplementary figures and images for: Streptomyces alboflavus RPS and Its Novel and High Algicidal Activity against Harmful Algal Bloom Species Phaeocystis globosa
Source: PLoS One. 2014 Mar 27;9(3):e92907. doi: 10.1371/journal.pone.0092907 (PMC3968035; doi:10.1371/journal.pone.0092907)

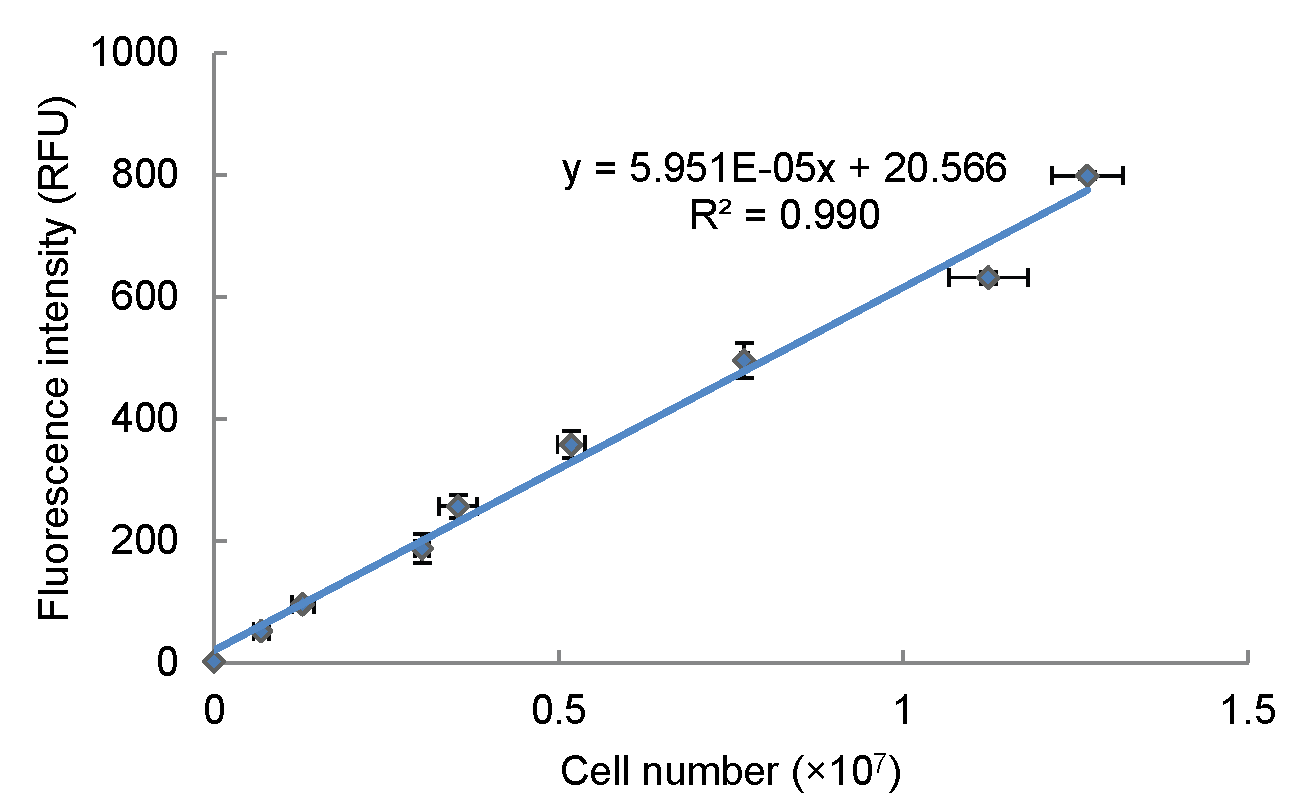

Supplement: Figure S1 — Linearity between the cell number of P. globosa and fluorescence intensity (RFU). Cell numbers were counted manually by optical microscope. RFU was measured at the excitation wavelength (λex) = 440 nm and emission wavelength (λem) = 680 nm (Spectra max M2, Molecular Devices Corporation). Data points: mean ± standard deviation of triplicate assays. The linear relationship was analyzed by linear regression (R2 = 0.990). (TIF) [file pone.0092907.s001.tif]

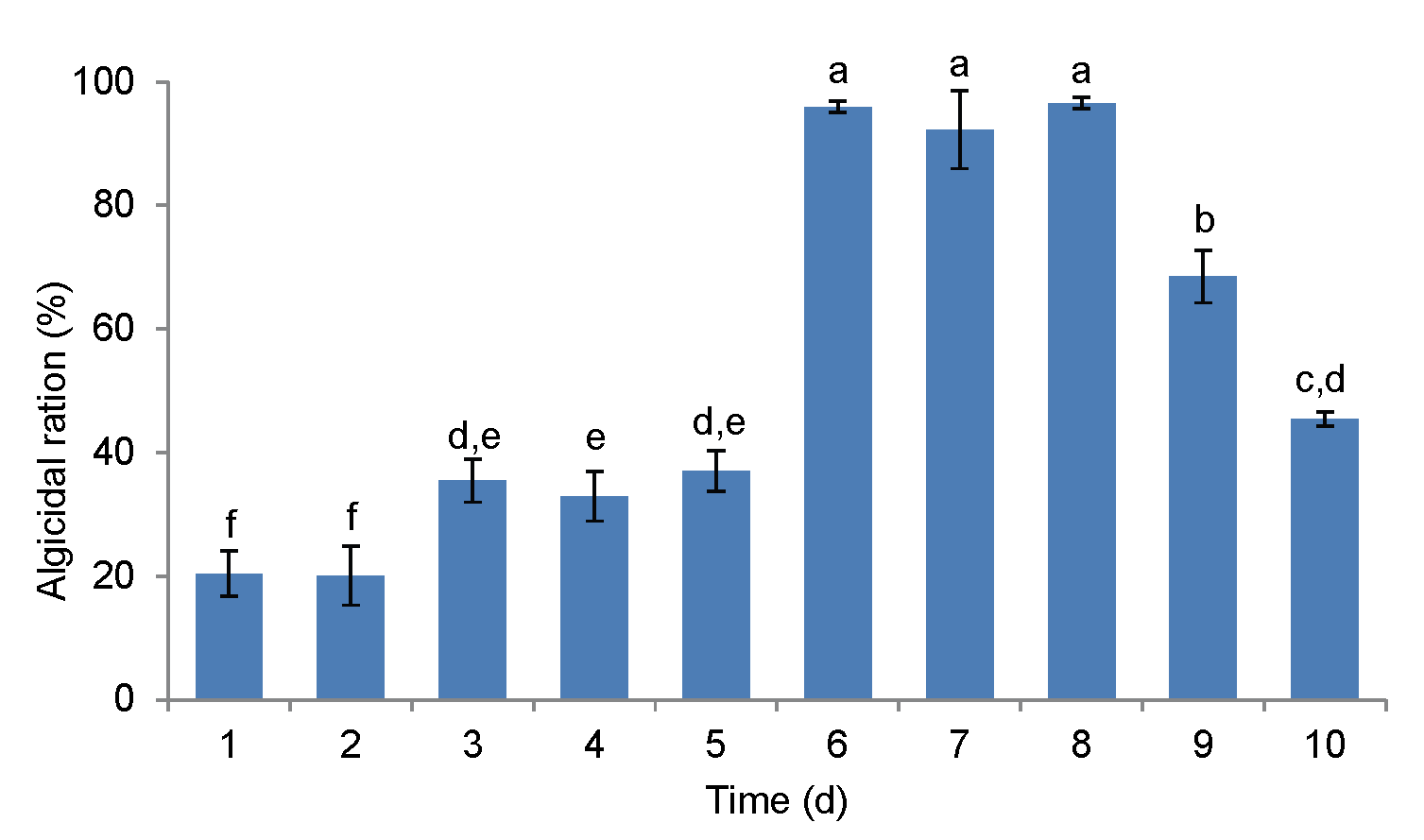

Supplement: Figure S2 — Effect of fermentation time on algicidal activity of RPS supernatant against P. globosa . Results showed that activities of the supernatants were higher at 6 d, 7 d and 8 d compared with those at early days, and decreased gradually over fermentation time, and 7d was chosen in this study. Data from RFU assay of algicidal effect (1∶10, v:v) of supernatant samples collected from RPS fermentation broth each day for 10 days. Data points: mean ± standard deviation of triplicate assays. Treatment means with different letters differed significantly (P<0.05) by analysis of one-way ANOVA. The wet weight and dry weight of the mycelia biomass in 100 mL fermentation broth for 7 d were approximately 0.77±0.06 g and 0.07±0.03 g, respectively. (TIF) [file pone.0092907.s002.tif]

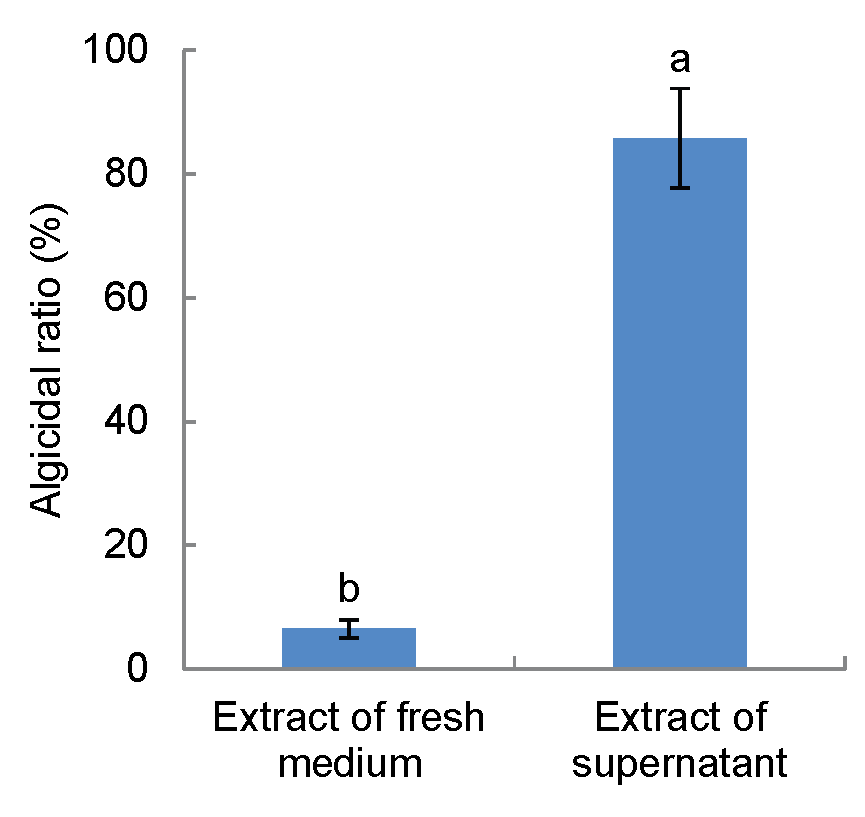

Supplement: Figure S3 — Algicidal activity comparison of ethyl acetate extract of fresh medium with that of fermentation supernatant. Data from RFU assay of algicidal effect of 10 μL extract added into 1.99 mL algal culture (the same ratio as the polarity test). Data points: mean ± standard deviation of triplicate assays. Since the algicidal ratio of extract of fresh medium is much weaker than that of supernatant extract (P<0.001) by analysis of one-way ANOVA, the weak effect can be ignored in this study. (TIF) [file pone.0092907.s003.tif]
