# Supplementary material for: Streptomyces alboflavus RPS and Its Novel and High Algicidal Activity against Harmful Algal Bloom Species Phaeocystis globosa
Source: PLoS One. 2014 Mar 27;9(3):e92907. doi: 10.1371/journal.pone.0092907 (PMC3968035; doi:10.1371/journal.pone.0092907)
Supplement: Table S1 — Cell concentration of each algal species (RFU approximately = 300) tested in this study. The value represents the average of three replicates. (DOCX) [file pone.0092907.s004.docx]

**Table S1.** Cell concentration of each algal species (RFU approximately =300) tested in this study. The value represents the average of three replicates.

| Phylum | Species | Real RFU | Concentration (cells mL^-1^) |
| --- | --- | --- | --- |
| Haptophyta | *P. globosa* | 307.41 | 2.42×10^6^ |
| Xanthophyta | *H. akashiwo* | 301.61 | 3.02×10^6^ |
| Chlorophyta | *C. autotrophica* | 281.54 | 6.31×10^6^ |
|  | *N. oceanica* | 312.56 | 7.57×10^6^ |
|  | *P. helgolandica* | 303.01 | 3.96×10^5^ |
|  | *P. subcordiformis* | 317.71 | 3.64×10^6^ |
|  | *D. salina* | 313.53 | 9.28×10^5^ |
|  | *Chlorella* sp. | 328.29 | 8.67×10^4^ |
| Chrysophyta | *D. inornata* | 273.69 | 3.18×10^6^ |
|  | *I. galbana* | 334.77 | 6.16×10^6^ |
| Pyrrophyta | *A. tamarense* | 289.35 | 5.55×10^5^ |
|  | *S. trochoidea* | 308.15 | 1.44×10^4^ |
| Bacillariophyta | *P. tricornutum* | 327.42 | 1.75×10^6^ |
|  | *A. japonica* | 295.16 | 5.60×10^5^ |
